# DISABLEMENT IN CONTEXT: DO SOCIAL NETWORKS MODERATE THE IMPACT OF INCREASING FUNCTIONAL IMPAIRMENT ON WELL-BEING?

**DOI:** 10.1093/geroni/igz038.2088

**Published:** 2019-11-08

**Authors:** William R McConnell, Robbie Dembo

**Affiliations:** 1 Florida Atlantic University, Boca Raton, Florida, United States; 2 Brandeis University, Boston, Massachusetts, United States

## Abstract

Older adults with disabilities face a higher risk of experiencing poor health and social isolation in later life. Prior research has shown that social factors such as supportive relationships can modify disablement trajectories and reduce the likelihood of negative outcomes. Although research has considered the functional benefits of relationships through examining mechanisms like social support provision, the effects of network structure on the disablement process are not well understood. This study examines multiple social network mechanisms to explain the link between disability, health, and social participation among older adults. We ask, do social networks characteristics mediate or moderate the effects of increasing functional impairment on health and social participation? We analyze longitudinal panel data from the National Social Life, Health, and Aging Project 2005 & 2010, including 2,261 adults aged 57-85. Respondents named 9,587 network members in 2005. We model indicators of health and social participation at 5-year follow-up using trajectories of functional impairment and social network characteristics. We find that larger, denser, and more supportive networks are associated with better health and more frequent social activity at follow-up. Furthermore, social network structure mediates the relationship between increasing functional impairment and health, but moderates the effect of impairment on social participation. For example, participants with more dense networks are more likely to maintain high social activity at follow-up, even at relatively high levels of impairment. This study demonstrates that functional impairments are not inherently disabling. Instead, personal and social resources can reduce the potential burdens of impairment in individuals’ lives.

